# Population‐Level Impact of Hypertension and Diabetes on Cognitive Domains: Insights from the Health and Aging Brain Study: Health Disparities

**DOI:** 10.1002/alz70860_099530

**Published:** 2025-12-23

**Authors:** Cellas A Hayes, Lubnaa B Abdullah, Joshua L Gills, Michelle C Odden, HABS‐HD

**Affiliations:** ^1^ Stanford University, Stanford, CA, USA; ^2^ University of North Texas Health Science Center, Fort Worth, TX, USA; ^3^ NYU Grossman School of Medicine, New York, NY, USA; ^4^ Stanford University, Palo Alto, CA, USA

## Abstract

**Background:**

Black individuals have a higher overall dementia risk than Non‐Hispanic Whites (NHW), potentially driven by a greater prevalence of vascular risk factors. This study examines the independent and interactive effects of race and cardiometabolic risk factors on cognitive performance and estimates the population‐level impact of hypothetical interventions using causal inference methods among Black and NHW participants.

**Method:**

Data from 1,098 NHW and 616 Black participants in the Health and Aging Brain Study: Health Disparities were analyzed. Multivariable regression models assessed interactions between race and cardiometabolic risk factors across four cognitive domains, controlling for demographics. Population intervention measures estimated the reduction in cognitive performance outcomes under the hypothetical elimination of risk factors, stratified by race. This method is similar to population attributable fraction calculations but allows for more flexible modeling.

**Result:**

Diabetes and hypertension were more prevalent among Black participants (26.3% and 80.4%) compared to NHW (13.5% and 58.7%, *p* < 0.001). Black participants consistently scored lower across all cognitive domains (‐0.34 to ‐0.57 points, *p* < 0.001). There were no significant interactions between race and diabetes or hypertension for any cognitive domain (*p* > 0.05). Hypothetical elimination of hypertension and diabetes was associated with larger improvements in cognitive performance for Black participants compared to NHW. For executive function, eliminating hypertension would increase scores by 0.07 points (95% CI: 0.01‐0.13) for Black participants and 0.05 (95% CI: 0.01‐0.09) for NHW. Language function could increase by 0.08 points (95% CI: 0.01‐0.14) among Black participants and 0.06 (95% CI: 0.01‐0.10) for NHW. Diabetes showed similar but more modest trends.

**Conclusion:**

Hypertension and diabetes disproportionately impact cognitive outcomes in Black participants, emphasizing their higher prevalence as key contributors to observed disparities in cognitive aging.

